# Psychological Gender and Emotional Intelligence in Youth Female Soccer Players

**DOI:** 10.1515/hukin-2015-0084

**Published:** 2015-10-14

**Authors:** Katarzyna Rutkowska, Józef Bergier

**Affiliations:** 1 Jozef Pilsudski University of Physical Education in Warsaw – Faculty of Physical Education and Sports in Biala Podlaska.; 2 Pope John Paul II State School of Higher Vocational Education in Biala Podlaska.

**Keywords:** females, youth sport, soccer, sport psychology

## Abstract

Many sports (for instance soccer) are stereotypically perceived as a male activity. Even so, more and more women decide to become competitive athletes. Since the theory of sport requires comprehensive explanations and the practice of sport needs clear guidelines, interdisciplinary studies into the nature of sport, including its psychological aspects, are necessary. Analysing the psychological profile of female soccer players, particularly those who are about to become professional athletes, can provide many interesting insights into the specific character of female youth sport and show where improvements can be made in athletic training programmes (especially in mental training). It is therefore important to study psychological gender that determines social behaviours and to analyse female athletes’ emotional intelligence. Emotional intelligence is defined as a set of emotional competencies that determine the effectiveness of human behaviours. Psychological gender and emotional intelligence have a significant effect on human adaptability and the efficiency of psychosocial functioning. This research was undertaken with the dual purpose of identifying the psychological gender and emotional intelligence of female soccer players. It involved 54 secondary-school girls, some of whom attended a sports class and others played on the Polish national team. The following tools were used to carry out the research: the Gender Assessment Inventory (IPP [This and the other acronyms derive from the Polish language]-developed by Kuczyńska) and the Emotional Intelligence Questionnaire (INTE; created by Jaworowska and Matczak). As shown by the analysis of the results, most female soccer players in the study were androgynous and the level of their emotional intelligence was significantly higher than in other participants. This also seems to point to their significantly greater adaptability. At the same time, the level of emotional intelligence in many players was average or low, which seems insufficient and calls for adequate intervention measures to be taken.

## Introduction

Soccer is perceived as a typically male domain, however, today its popularity is increasing among women as well (Jeanes, 2011; Soroka and Bergier, 2011). Female soccer players must develop and carry out their own models of femininity, alternative to traditional roles performed by women in particular cultures. The course of this process largely depends on one’s gender identity and the subjective perception of what it means to be female. Other important factors are the climate that predominates in the community, and the norms along with standards that female players recognize and live by (Jeanes, 2011). Naturally, one of the key variables is socialisation and the models of female or male roles that are acquired in its course. The influence exerted by parents, guardians, and coaches, as well as by school, the media, and peers outside the athletic community should also be taken into account, as it has been observed that training for soccer, somewhat boyish behaviour, the need to cope with physical demands (endurance, strength), and aggression on the pitch are sometimes perceived as inappropriate, useless and unnatural for girls, or as crossing the line of femininity. Many girls faced with entrenched stereotypes give up competitive sport (Guillet at al., 2006; Stirling et al., 2011).

The above context highlights the importance of research into the special nature of female sport with interdisciplinary studies. One variable which needs to be stressed is psychological gender, which is the effect of culturally-formed concepts of being a woman and/or a man on self-perception, an inclination to process information about „myself” through stereotypical gender models, or the acceptance of specific male or female attributes of personality and the readiness to take advantage of them (Bem, 1974, 1981; Kuczyńska, 1992).

Studies with female players have shown that those scoring higher on the „masculinity” scale tend to perceive themselves as more competent and more determined, meaning that they are also more inclined to continue their involvement in sport. Authors have also observed that the masculine or androgynous players discontinue their sport careers relatively less frequently (Guillet et al., 2000, 2006).

Soccer coaches recommend giving more attention to improving the playing culture of young players and competencies such as self-awareness, stress-coping capabilities, and intelligence (including emotional intelligence). Their suggestions are consistent with the general need to humanize sport already at the youth level. Efforts to create an environment that would make it easier for youth athletes to move smoothly towards competitive sport and adjust optimally to its demands should be accompanied by comprehensive research into psychosocial factors (Mills et al., 2012). Special attention should be given to the development and improvement of emotional intelligence, which is defined as individual’s meta-ability to freely use their emotions and emotional knowledge in acting and thinking (Goleman, 1995; Jaworowska and Matczak, 2001). Emotional intelligence is recognised as a major determinant of effective adaptation to varying conditions and of success in professional, social or sports life combined with the satisfaction that one has reached their goals (Goleman, 1995; Meyer and Fletcher, 2007; Lane et al., 2010; Chan and Mallett, 2011).

Taking into consideration specific characteristics of female sport, an interesting matter to analyse is psychological gender of female athletes who choose sports stereotypically perceived as male. An in-depth analysis of personality differences (including emotional intelligence) between female athletes representing different types of psychological gender may be interesting for coaches as well. The knowledge of the psychological profile of a player can be used to better understand her, to improve communication and training effectiveness, and to encourage the player to be more committed to developing as an athlete and a human. Lastly, optimal support can be offered in specific situations, such as when one has to decide whether to continue a sports career.

## Material and Methods

The purpose of the present study was to identify psychological gender of the selected female soccer players and to assess their emotional intelligence. The study was carried out with a sample of 54 girls (age: 16.85 ± 0.83) who were soccer players (training experience: 5.41 ± 2.57). Most of the participants (37) attended a sports class at a secondary school (almost 70%). The other 17 played on the Polish national youth soccer team.

The participants were surveyed using two research tools: the IPP (Gender Assessment Inventory) and the INTE (the Emotional Intelligence Questionnaire). The IPP takes advantage of two scales – femininity and masculinity – to analyse two aspects of psychological gender and therefore allows four types of gender to be identified (in a group of females) at the same time: 1/ sex-typed individuals – feminine females, 2/ androgynous individuals ranking high on both scales, 3/ non-sex-typed individuals who are low on both scales, 4/ cross-sex-typed individuals – masculine females (Kuczyńska, 1992). Since the primary purpose of the original questionnaire was to survey people older than 18 years of age, in the research it was experimentally modified to make it suitable for younger participants. Terms such as “experimenting in sexual life” on the masculinity scale and „coquettish” on the femininity scale were removed. Consequently, the original scoring system was modified towards the research objectives. The other tool, a Polish version of the Emotional Intelligence Questionnaire [The questionnaire was developed by N. S. Schutte, J. M. Malouff, L. E. Hall, D. J. Haggerty, J. T. Cooper, Ch. J. Gloden, L. Dornheim (Schutte at al., 1998)], serves to assess emotional intelligence which is defined as person’s ability to recognize their emotions and to use the knowledge in acting and thinking. Regarding this tool, in statistical analysis the original scoring system was used, which allows raw scores to be converted based on sten norms for the secondary school pupils (Jaworowska and Matczak, 2001).

## Results

Psychological gender of the selected female soccer players was determined by analysing scores they obtained with the modified IPP questionnaire. Almost 65% of the participants were androgynous (N=35), meaning they ranked high on both the femininity and masculinity scales. The mean values calculated for the whole sample (femininity M=50.50, SD=7.02; masculinity M=48.81, SD=7.41) also pointed to the androgynous gender type being in majority (N=35). The least frequent in the sample (N=3) were the non-sex-typed players. The other participants were “masculine females” (N=9) and “feminine females” (N=7).

The levels of participants’ emotional intelligence were established using the INTE questionnaire. The raw mean M=122.35, SD=13.46 and the converted mean M=4.76, SD=2.02 suggest that the participants had an average level of emotional intelligence (within the lower limit). An additional analysis of the converted scores showed how the shares of the three categories [Low score (1–3 stens), average score (4–7 stens), high score (8–10 stens)], of scores were distributed. More than half of the participants had an average level of emotional intelligence (N=29) and every third low (N=17). In eight participants, the level of emotional intelligence was high (one of them achieved a score corresponding to 10th sten).

Within analysis, the levels of emotional intelligence (**RD** – raw data, **CD** – converted data) characterising androgynous players and those identified as masculine, feminine and non-sex-typed that altogether represented the category “other gender” were also compared (Table 1).

The analysis showed the androgynous participants to have a significantly higher level of emotional intelligence (the raw score and the converted INTE score provided the same conclusion).

The Spearman’s correlation coefficients (Table 2) revealed strong correlation between the level of intelligence and two basic dimensions of psychological gender – femininity and masculinity.

## Discussion

The results yielded by the study presented above are similar to those obtained by other authors who investigated psychological gender of female athletes (Soroka and Bergier, 2011; Wiliński, 2012). The androgynous female athletes formed the most numerous group. According to the literature on this subject, this group shows the best adaptability (Kuczyńska, 1992; Soroka and Bergier, 2011). Its members are capable of functioning effectively in different life situations and the psychological costs they pay are relatively low. This type of psychological gender may also allow the female players to be optimally feminine in the stereotypically male world (Wiliński, 2012), thus, making it more probable that they will maintain their interest in sport and will continue careers as athletes (Guillet et al., 2000, 2006). To find out how female athletes can remain female, in-depth interdisciplinary studies are necessary, conducted in order to develop precise guidelines for a coaching programme addressing the needs of women practicing sports stereotypically perceived as male domains. One of the programme’s objectives should be to create conditions enabling female athletes to perform female roles in an optimal and satisfying manner (i.e. without incurring unnecessary psychosocial costs).

The higher capacity for adaptation of androgynous athletes has been confirmed by the results of other studies (Mroczkowska, 2005), which also point to these athletes having greater emotional balance. Higher emotional intelligence identified during this research in the androgynous players (and the significant correlation between femininity and masculinity that represent the two dimensions of psychological gender) provides the same conclusion. However, the level of emotional intelligence was found to be average compared with the female population at large and therefore seems insufficient. The scores achieved by the other participants (i.e. those not in the androgynous group) show the necessity of intervention. Considering the present knowledge about emotional intelligence, the specific nature of team games and demands that the female athletes have to cope with, it seems advisable that this type of intelligence is developed in athletes. When designing the psychopedagogic measures, the development and improvement of elements of social competencies other than emotional intelligence alone are also worth considering.

With the benefits of higher emotional intelligence presented in this article, the androgynous female athletes could better identify and fulfil their model of psychological gender, have more effective and satisfying lives, and realise themselves more fully within and outside sport. Some authors stress the importance of the purposeful development and improvement of emotional intelligence viewed as a universal meta-ability (Meyer et al., 2007; Lane et al., 2010; Sadri, 2013) and there are also proposals to enhance other dimensions of the human potential (Subotnik and Edmiston, 2010). Even if these proposals do not directly relate to sport, they can serve as a valuable inspiration and, after appropriate modifications, as a component of the psychosocial skills training dedicated to female soccer players.

Research results indicate that athletes in team sports have more developed emotional intelligence compared with their untrained peers. The explanation of this finding is sought in the demands of sport, mainly in the need to control emotions and to manage them optimally in different situations (Zamanian et al., 2011). Although in this study the male and female soccer players were not compared for the levels of emotional intelligence, other investigations into female sport have showed that female wrestlers have less developed emotional intelligence than their untrained peers and that they do not differ significantly in this respect from young male wrestlers (Rutkowska and Gierczuk, 2012). It would therefore be interesting to conduct another study comparing the levels of emotional intelligence between female soccer players and untrained girls, and between male soccer players and untrained boys. This type of analysis could generate interesting information enabling the modification and improvement of training processes, as well as helping increase the scope of psychological training dedicated to young female soccer players.

It is natural and obvious that men’s soccer and women’s soccer are dissimilar. Since male and female players differ in respect to their physical and psychosocial abilities, the tactics and technique should be defined accordingly. For some authors who view soccer as an all-male sport, women’s achievements are manifestations of feminism. Hjelm (2011) argues, however, that female soccer deserves careful analysis and more balanced judgment. Those who share this approach have already started an interesting debate. Do coaches sufficiently address the differences between men’s and women’s soccer? Is female sport recognised well enough for coaches to be able to respond to the athletes’ specific demands? And, finally, are coaches sensitive enough to the issue of female athletes and their needs? In some circles these questions are raised quite frequently and suggestions are put forward to modify professional education for coaches. Those who advocate this approach argue that coaches in female soccer who want to have good communication with individual players and the team should have „scenarios” for handling different situations that typically occur in female sport, but more importantly, they should be able to understand the mentality of female athletes (Milyak and College, 2010). Cultural and religious aspects are also important. The sense of identity and identification with certain social models of gender seem to be crucial. The relationships observed in sport seem to be multidirectional: changes in the perception of sport as an element of culture may be induced by social changes or entail them (Koh, 2003; Perets et al., 2011; Ratna, 2011). Therefore, specific characteristics of women’s sport cannot be discussed without understanding a broad context that also includes special characteristics of social gender stereotypes.

The authors presented the results of research carried out with the Polish version of the research tools. The questionnaire INTE is a Polish adaptation of the questionnaire presented in the literature (Schutte at al., 1998), elaborated with reference to the classical model of emotional intelligence (Salovey and Mayer, 1990). The inventory IPP is an originally Polish tool, based on a well-known model of the Gender Schema Theory (Bem, 1981). The authors are aware that necessity of using Polish language versions of tools (with reference to the Polish-language textbooks) can provide a kind of restriction, especially when attempting to compare the results of own investigations with the results of authors from other countries. However, it can be an inspiration to realize cross-national research.

The discussion about the future shape of female soccer is emerging in Polish soccer as well. A wide-ranging discussion needs an appropriate knowledge base, including comprehensive reports on studies (encompassing psychological variables) into the special characteristics of women’s soccer in Poland. The authors of this research hope that the presented results will help advance the discussion and encourage new research. The problems of the contemporary women’s sport require appropriate solutions, however, for these to be developed effective cooperation between scientists and practitioners is necessary.

## Conclusion

The results of the present study showed that psychological gender of most female soccer players in the sample was androgynous and that these players had a higher level of emotional intelligence. The majority of the players, over 85%, had an average or low level of emotional intelligence. The obtained results show necessity of further analysis of the psychological profile of female soccer players, particularly of those who aspire to become professional athletes. The results also indicate that targeted actions are necessary, focusing on both athletic training and psychological formation. They also give one more argument for cooperation between players and coaches and a sport psychologist from the youth sport level forward. Accurate analysis (involving also the identification of psychological gender), psychosocial training (to develop and enhance emotional intelligence, among other skills), various educational, corrective and preventive measures could raise training quality and effectiveness in women’s soccer, and consequently support the athletes in their pursuit of sports career. Through cooperation with a psychologist young female soccer players can create an optimal vision of their development as athletes and humans.

## Figures and Tables

**Table 1:** Psychological gender vs the level of emotional intelligence in selected female soccer players

Level of emotional intelligence	Psychological gender		Inter-group comparison

	Androgynous N=35 M; *SD*	other gender* N=19 M; *SD*	Mann-Whitney U-test, *p*
			
Emotional intelligence (**RD**)	126.31; *13.91*	115.05; *9.03*	179.00; *p*≤*0.005*
Emotional intelligence (**CD**)	5.37; *2.14*	3.63; *1.12*	172.50; *p*≤*0.005*

* feminine, masculine, non-sex-typed

**Table 2:** Correlations between femininity and masculinity and the level of emotional intelligence in selected female soccer players

Level of emotional intelligence	Psychological gender	
	
	femininity	masculinity
		
Emotional intelligence (**RD**)	.36*	.34*
Emotional intelligence (**CD**)	.36*	.37*

* p≤0.01
